# Molecular determinants of cesium- and glycine-dependent glycine receptor activation

**DOI:** 10.1038/s41598-025-20807-y

**Published:** 2025-09-23

**Authors:** Magnus Harnau, Elina Zeller, Steffen Fricke, Jochen C. Meier

**Affiliations:** Division Cell Physiology, Technical University Braunschweig, Spielmannstr. 7, 38106 Braunschweig, Germany

**Keywords:** Ion channels in the nervous system, Molecular neuroscience

## Abstract

The neurotransmitter receptor for glycine (GlyR) is a ligand-gated chloride-permeable ion channel in the central nervous system, and dysregulation of this channel is associated with diverse neurological disorders. Cesium (Cs^+^) is an agonist of GlyRs, and recent atomistic molecular dynamic simulations suggested amino acids D141, E192, and D194 with negatively charged side chains as possible binding sites for Cs^+^. To test this hypothesis, we mutated these positions to code for alanine with a neutral hydrophobic side chain or lysine with a positively charged side chain. The correspondingly mutated GlyR channels were expressed in HEK293T cells and analyzed using whole-cell patch clamp electrophysiology. The results show that D141 mutations had no considerable effect on GlyR currents, E192K prolonged desensitization and D194 turned out to be critical for general (glycine- and Cs^+^-dependent) GlyR activation, which was not due to differentially reduced cell surface expression of all the investigated GlyR mutants. Thus, E192 and D194 are critically involved in cell surface GlyR activation by Cs^+^ and glycine.

**Supplementary Information:**

The online version contains supplementary material available at 10.1038/s41598-025-20807-y.

## Introduction

The neurotransmitter receptor for glycine (GlyR) is key to regulation of respiration and neuromotor coordination, hearing, neurogenesis, cortical cell migration, cognition and nociception as it is expressed in neurons of the spinal cord, brainstem, cortex, hippocampus and even in non-neuronal cells^1–3^. Dysregulation of GlyR function can thus result in several diseases including neurological disorders such as autism, epilepsy and chronic inflammatory pain as well as cardiovascular and respiratory dysfunction. Hence, research focusing on the structure-function relationship of GlyRs is required for a better understanding of the diverse causes of associated diseases.

The GlyR is a chloride-permeable receptor and part of the pentameric, ligand-gated Cys-loop ion channel family. Depending on the chloride concentration, GlyR activation results in inhibition or excitation at the synapse. GlyRs consist of five subunits of either α1–4 or together with a β subunit, which functions as anchor to the postsynaptic matrix protein gephyrin. The receptor has N- and C-terminal extracellular domains separated by four transmembrane-spanning (TM) regions^4^. It was proposed that GlyR gating happens via the movement of TM2 and that the flanking loops, such as the F-loop (β8-β9), Cys-loop (β6-β7), β1-β2 loop, and the β10 strand which precedes the TM1 helix, serve as connections from the extracellular domain to the TM2-TM3 linker. In this way, the conformational change after binding of glycine at the interface between two subunits is transitioned to open the internal pore^5–7^.

We previously investigated the effect of RNA-editing of GlyRs on the potency of the agonists glycine, taurine, and GABA^8,9^. C-to-U RNA-editing results in amino acid substitution from proline to leucine at position 185 in the mature signal peptide-processed GlyR α1 and α3 proteins while the edited position corresponds to 192 in mature GlyR α2^8–10^. In all cases, RNA-editing of GlyRs results in a gain-of-function possibly contributing to the diverse neuropsychiatric symptoms of temporal lobe epilepsy^11–13^.

The recently identified GlyR agonists Cs^+^ and NH_4_^+^ also showed increased potency at RNA-edited GlyRs^14,15^. Atomistic molecular dynamic simulations were utilized to predict possible Cs^+^ binding domains in the GlyR and revealed two potential sites in the extracellular receptor domain. The first site is found slightly above the glycine binding site and the more prominent site comprises D141, E192, and D194 flanking the F-loop or β8-β9 loop^15^. Interestingly, the RNA-editable position P185 is located within this region^9^. While D141 is part of the Cys-loop that is conserved in the receptor family, E192 and D194 are located on the β-sheet 9. In addition to F159, Y202, T204 and F207 from one subunit and R65 and S129 from the neighboring subunit that directly participate in glycine binding^6^, this loop structure between β-sheets 8 and 9 emerges as critical determinant of the signal transduction after glycine binding^16^.

Divalent cations, such as Zn^2+^ and Cu^2+^, are also known to bind the GlyR and exhibit modulatory effects on glycine-evoked currents^17–19^. Zn^2+^ is a biphasic modulator that either inhibits or potentiates GlyR activation depending on its concentration and by binding to two different sites of the receptor. The binding moiety described for its potentiating effect is assumed to consist of E192, D194 and H215. The E192A and D194A mutations in GlyR α1 ablated the potentiating effect of Zn^2+^ and GlyR D194A had a threefold higher EC_50_, indicating less potency. For E192 the effect was interpreted as more indirect, possibly through perturbation of the backbone structure^20^. However, GlyRs are not directly activated by Zn^2+^, and it was originally proposed that its potentiating effect was due to slowing agonist dissociation^21^, and more recently due to intermediate conformations in the gating cycle^19^. In addition, E192 was also found to be part of the Cu^2+^ binding site, but interestingly Cu^2+^ only inhibits the glycine-evoked GlyR currents^18^.

In this study, we mutated the positions D141, E192, and D194 to code for the amino acid alanine with a neutral hydrophobic side chain or lysine with a positively charged side chain. Whole-cell patch clamp electrophysiology using transfected HEK293T cells revealed no significant effect of D141 mutations on GlyR activation by Cs^+^ or glycine. However, the E192K channel exhibited a prolonged desensitization and D194 mutations strongly reduced the amplitude of both glycine- and Cs^+^-evoked GlyR currents, which was not due to impaired GlyR surface expression. Thus, our study adds molecular details to the complex structure-function relationships in GlyRs as they reveal E192 and D194 on β-sheet 9 as critical determinant of either GlyR desensitization or activation by Cs^+^ and glycine.

## Methods

### Molecular cloning and expression constructs

The mutations in the glycine receptor (GlyR) α3L (NCBI accession number NM_080438.4) P185L-expressing construct were introduced by PCR with primers carrying the corresponding mutations leading to D141A, D141K, E192A, E192K, D194A or D194K conversion. HA-epitope tagged constructs used for analysis of GlyR surface expression were generated likewise by inserting the epitope tag YPYDVPDYA between the second and third amino acid position in the mature signal peptide-cleaved GlyR α3, which is a location suitable for GlyR surface staining using epitope tags^22,23^. Ligation was achieved with the NEBuilder Hifi DNA Assembly Master Mix (New England Biolabs, #E2621). Transformation into chemically competent *Escherichia coli* XL1blue by heat shock and subsequent preparation of plasmids with Monarch Spin Plasmid Miniprep Kit (New England Biolabs, #T1110L) and PureLink™ HiPure Plasmid Midiprep Kit (Invitrogen™, #K210005) led to the constructs used in this study. The primer production and the plasmid sequencing were carried out by Eurofins Genomics Germany GmbH. The GlyR transcription was driven by a CMV promotor and EGFP was co-expressed via an IRES.

### HEK293T culture and transfection

The human embryonic kidney (HEK) 293T cells (DSMZ, ACC 635) were maintained at 37 °C and 5% CO_2_ in T25 flasks containing 5 mL DMEM (Gibco™, #41965-062) with 10% fetal calf serum (Gibco™, #10500064) and 1% penicillin/ streptomycin (Gibco™, #15140122). Cells were passaged twice a week when nearly reaching confluency and seeded onto 35 mm culture dishes to reach ca. 800k cells for the subsequent transfection with FuGENE HD transfection reagent (Promega, #E2311). The transfection was accomplished by following the protocol of the manufacturer and using 1 µg of expression vector DNA. After incubation over-night, the cells were enzymatically detached and seeded on 13 mm glass coverslips (VWR, #631–1578). Prior to seeding, the coverslips were pre-treated with 10 M NaOH for 3 h at 100 °C, sterilized at 180 °C for 8 h and coated with 0.5 mg/mL poly-L-lysine hydrobromide (Sigma Aldrich, #P2636) for 3 h at 37 °C. After sufficient attachment of the cells to the surface of the coverslips (1–2 h), whole-cell patch clamp experiments were performed. For the GlyR surface staining, 10,000 HEK293T cells were seeded on lysine-coated coverslips in 24 well plates. Transfection was performed on the next day with FuGENE and 0.25 µg plasmid.

### Electrophysiology

The coverslips with the GlyR expressing HEK293T cells were placed in an open imaging chamber under a Nikon Ts2R microscope and whole-cell voltage clamp was executed using an EPC 10 amplifier and Patchmaster software (HEKA). Cells were used for the experiment when EGFP expression was confirmed with a pE-4000 illumination system (CoolLED) and a Nikon sCMOS pco.edge camera. Borosilicate glass capillaries (World Precision Instrument, #1B150F-4) were pulled with a PC10 puller (Narishige) and their resistances lay between 4 and 8 MΩ when filled with the following intracellular solution (in mM): CsCl (130), NaCl (5), CaCl_2_ (0.5), MgCl_2_ (1), EGTA (5) and HEPES (30); pH = 7.2 (CsOH). Standard extracellular solution (ES) contained (in mM): NaCl (140), KCl (5), MgCl_2_ (1), CaCl_2_ (2), HEPES (10) and glucose (10); pH = 7.4 (NaOH). The N-methyl-D-glucamine (NMDG^+^) extracellular solution ensured that no monovalent cations were present and contained (in mM): NMDG^+^ (150), MgCl_2_ (1), CaCl_2_ (2), HEPES (30) and glucose (10); pH = 7.4 (HCl). Dose-response curves were obtained using various concentrations of Cs^+^ prepared by mixing with NMDG solution (in mM): CsCl (0.5, 5, 50, or 150), MgCl_2_ (1), CaCl_2_ (2), HEPES (10) and glucose (10); pH 7.4 (CsOH). Dose-response curves were also obtained using various concentrations of glycine (in µM: 0.01, 1, 10, 100, 500, 1000) in NMDG^+^. To ensure rapid fluid exchange a gravity-driven perfusion system equipped with a perfusion pencil with a 360 μm tip (AutoMate Scientific, #04-08-250) and a suction pump were utilized. The HEK293T cells were opened in presence of ES, and NMDG solution was applied at the beginning of the experiment and in between the various glycine and Cs^+^ solutions. The voltage clamp holding potential was -50 mV and the series resistances (R_s_) were monitored by 50 ms long -5 mV voltage pulses every 5 s. The patch data were recorded at 20 kHz and filtered with a 2.8 kHz Bessel filter. All experiments were carried out at room temperature.

### GlyR surface staining and confocal microscopy

One day post-transfection, the GlyR expressing HEK293T cells on lysine-coated coverslips were stained and fixed in a wet chamber. Initially, the cells were washed with 0.1% PBS-gelatin and then incubated with 50 µL primary antibody (chicken anti-HA, 1:200) for 15 min at 37 °C in the incubator. After washing twice with PBS-gelatin, the cells were fixed with 4% PFA/sucrose solution for 15 min. Following this, washing was repeated thrice and the coverslips were treated with 50 µL secondary antibody (anti-chicken IgY-Cy3, 1:200) for 45 min. Finally, the cells were rinsed two times with PBS-gelatin, three times with PBS, mounted with Vectashield containing DAPI and then sealed with nail polish. Fluorescence imaging was conducted with the Stellaris 5 confocal laser scanning microscope by Leica Microsystems. Images were recorded in 8-bit with 1024 × 1024 resolution and fluorescence was excited at 552 nm for Cy3, 489 nm for EGFP and 405 nm for DAPI. Emission was recorded in the interval of 557–650 nm for Cy3, 494–550 nm for EGFP and 420–460 nm for DAPI, respectively. Laser intensity and gain settings were kept constant and recording was performed with 2x average to reduce background fluorescence.

### Data analysis and statistics

Electrophysiology data analysis was performed offline with IGOR Pro software and a custom written tool by M. Semtner^24^. The peak amplitudes were noted, the baseline subtracted and Cs^+^-evoked currents were normalized to the maximum current amplitudes in response to 0.1 mM glycine. For the glycine dose-response curve, current amplitudes were normalized to the glycine concentration that elicited the highest maximum current amplitude. For the desensitization analysis, the exponential decline of the current amplitude in response to 1 mM glycine or 150 mM Cs^+^ was fitted in IGOR Pro and the time from peak to the end of plateau was measured to obtain the time constant of desensitization (τ). Cells with R_s_ higher than 35 MΩ were excluded. Statistical analysis was performed using the Graph Pad Prism software. The absolute and normalized amplitudes were examined by repeated-measures two-way ANOVA test with the recommended Geisser-Greenhouse correction and Tukey´s multiple comparison test with individual variances computed for each comparison. Dose-response-dependent non-linear regression as well as EC_50_ and Hill coefficient calculation were also performed using Graph Pad Prism.

The fluorescence images of the GlyR surface staining were analyzed by comparison of the whole image Cy3 fluorescence in ratio to the number of transfected cells. In advance, the background of the Cy3 channel, which displayed the HA-tagged GlyRs, was subtracted in ImageJ. Therefore, the rolling ball tool with a radius set to 50 μm was utilized and in addition a general value of 10 was subtracted. This was determined by measuring the unspecific fluorescence as observed in the negative control, the EGFP-expressing HEK293T cells. Finally, the data were normalized to the results of the non-mutated GlyR α3L^185L^. Statistical analyses were performed using the Kruskal-Wallis test with Dunn´s multiple comparison test because the fluorescence ratios were not normally distributed. Statistical differences are indicated with asterisks: *p < 0.05, **p < 0.01, ***p < 0.001, and ****p < 0.0001.  

**Table:** Key resource table.

Reagent or resource	Source	Identifier
Antibodies		
Chicken polyclonal anti-HA antibody	BETHYL Lab. Inc., Montgomery, TX, USA	Cat.# A190-106 A
Donkey anti-chicken IgY-Cy3 antibody	Dianova by Biozol Diagnostica, Hamburg, Germany	Cat.# 703-165-155
Chemicals, peptides, and recombinant proteins		
Calcium chloride dihydrate	Carl Roth	Cat.# T885.2
Cesium chloride	Carl Roth	Cat.# 8627.1
Cesium hydroxide	Fluka	Cat.# 21000-10G-F (tested with NMR)
d-Glucose monohydrate	Sigma-Aldrich	Cat.# G8270-1 kg
Dulbecco´s Modified Eagle Medium (DMEM)	Gibco™	Cat.# 41,965,039
EGTA	AppliChem	Cat.# 67-42-5
Gelatin	VWR Normapur	Cat.# 24350.262
Glycine	Biomol	Cat.# 04943.1
HEPES	Carl Roth	Cat.# 9105.4
Heat inactivated Fetal Bovine Serum	Gibco™	Cat.# 10,500,064
Hydrochloric acid 37%	VWR Normapur	Cat.# 20252.290
Magnesium chloride hexahydrate	Carl Roth	Cat.# A537.4
N-Methyl-d-glucamine (NMDG)	Sigma-Aldrich	Cat.# M2004-500 g
Paraformaldehyde (PFA)	Sigma-Aldrich	Cat.# P6148-500G
Penicillin/Streptomycin	Gibco™	Cat.# 15,140,122
Phosphate-buffered saline (10x PBS)	Gibco™	Cat.# 14 200 − 067
Poly-l-lysine hydrobromide	Sigma-Aldrich	Cat.# P2636
Potassium chloride	VWR Normapur	Cat.# 26764.260
Sodium chloride	Carl Roth	Cat.# P029.2
Sodium hydroxide	VWR Normapur	Cat.# 28244.262
Sucrose	Carl Roth	Cat.# 9097.1
Trypsin/EDTA (1x)	Sigma-Aldrich	Cat.# T2601
Critical commercial assays		
FugeneHD	Promega	Cat.# E2311
Experimental models: cell lines		
HEK293T	DSMZ	ACC 635
Recombinant DNA		
GlyR α3L-185 L (C-terminal IRES-dependent EGFP-expression)	Corresponding to NCBI NM_080438.4	Lab ID: 54.131.1
GlyR α3L-HA-185 L (C-terminal IRES-dependent EGFP-expression)	Corresponding to NCBI NM_080438.4	Lab ID: 58.170.1
GlyR α3L-185 L-D141A (C-terminal IRES-dependent EGFP-expression)	Corresponding to NCBI NM_080438.4	Lab ID: 54.168.1
GlyR α3L-HA-185 L-D141A (C-terminal IRES-dependent EGFP-expression)	Corresponding to NCBI NM_080438.4	Lab ID: 64.251.1
GlyR α3L-185 L-D141K (C-terminal IRES-dependent EGFP-expression)	Corresponding to NCBI NM_080438.4	Lab ID: 54.168.3
GlyR α3L-HA-185 L-D141K (C-terminal IRES-dependent EGFP-expression)	Corresponding to NCBI NM_080438.4	Lab ID: 64.251.2
GlyR α3L-185 L-E192A (C-terminal IRES-dependent EGFP-expression)	Corresponding to NCBI NM_080438.4	Lab ID: 54.177.1
GlyR α3L-HA-185 L-E192A (C-terminal IRES-dependent EGFP-expression)	Corresponding to NCBI NM_080438.4	Lab ID: 64.251.3
GlyR α3L-185 L-E192K (C-terminal IRES-dependent EGFP-expression)	Corresponding to NCBI NM_080438.4	Lab ID: 54.177.3
GlyR α3L-HA-185 L-E192K (C-terminal IRES-dependent EGFP-expression)	Corresponding to NCBI NM_080438.4	Lab ID: 64.251.4
GlyR α3L-185 L-D194A (C-terminal IRES-dependent EGFP-expression)	Corresponding to NCBI NM_080438.4	Lab ID: 54.179.1
GlyR α3L-HA-185 L-D194A (C-terminal IRES-dependent EGFP-expression)	Corresponding to NCBI NM_080438.4	Lab ID: 64.251.5
GlyR α3L-185 L-D194K (C-terminal IRES-dependent EGFP-expression)	Corresponding to NCBI NM_080438.4	Lab ID: 54.179.2
GlyR α3L-HA-185 L-D194K (C-terminal IRES-dependent EGFP-expression)	Corresponding to NCBI NM_080438.4	Lab ID: 64.251.6
pEGFP-N1	Clontech, NCBI: U55762.1	Lab ID: 64.186.1
Software and algorithms		
IgorPro 6.3.7.2	WaveMetrics Inc.	www.wavemetrics.com
ImageJ	^25^	https://imagej.net/ij/download.html
Patchmaster 2 × 90	HEKA Electronik GmbH	https://www.heka.com/downloads/downloads_main.html
Prism 8	GraphPad	https://www.graphpad.com/
VMD	^26^	https://www.ks.uiuc.edu/Research/vmd/
Other		
Kwik-Fil borosilicate glass capillaries	World Precision Instruments	Cat.# 1B150F-4
Vectashield with DAPI	VectorLabs	Cat.# H-1200
13 mm diameter glass coverslips	VWR	Cat.# 631–1578
8 channel perfusion pencil	AutoMate Scientific, Inc.	Cat.# 04-08-360

.

## Results

We recently showed that GlyRs and particularly the RNA-edited 185L variant are activated by Cs^+^. Atomistic molecular dynamic simulations on GlyR α3 revealed a possible binding site consisting of D141, E192 and D194^15^. Here, we addressed the hypothesis that these positions are involved in Cs^+^-dependent GlyR activation and converted these negatively charged amino acids into neutral alanine or positive lysine. After expression in HEK293T cells we performed whole-cell patch clamp experiments and investigated the impact of these mutations on glycine and Cs^+^-evoked GlyR currents.

**Fig. 1:**
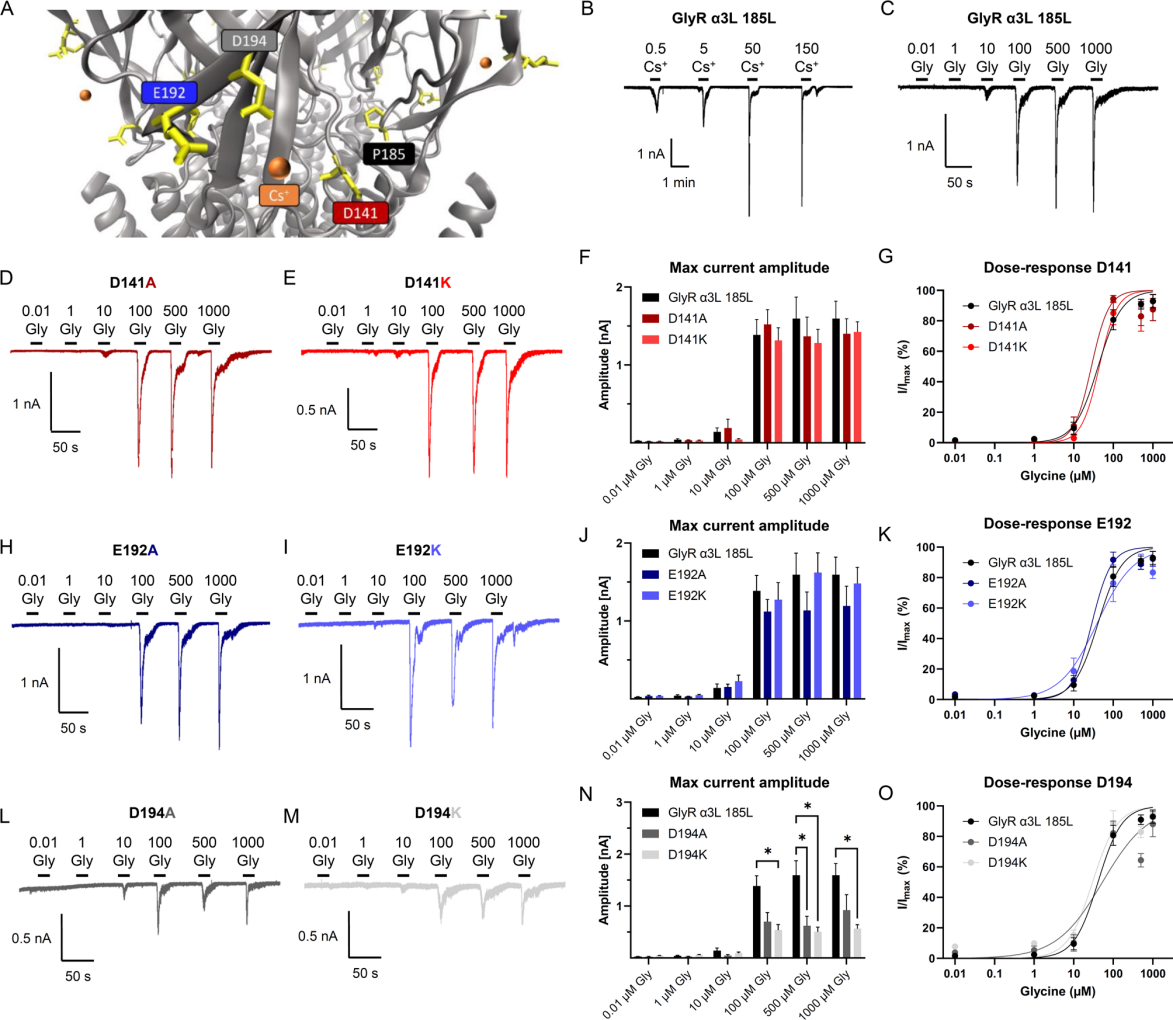
Structure-function relationships of GlyR α3L^185L^ and D141, E192, and D194 mutants with regard to glycine-dependent receptor activation. (**A**) Prediction of GlyR activation-relevant Cs^+^-binding sites D141 (red), E192 (blue), and D194 (grey) and the P185 position (black). Cs^+^-ions are displayed in orange. (**B**) Representative patch clamp recording from a HEK293T cell expressing non-mutated GlyR α3L^185L^ subjected to various concentrations of Cs^+^ (in mM). (**C**–**E**) Representative patch clamp recordings from HEK293T cells expressing non-mutated (**C**) and mutated GlyR α3L^185L^ D141A/K (**D**, **E**) subjected to various glycine concentrations (in µM). (**F**, **G**) Quantification of the absolute maximum current amplitudes (**F**) and glycine dose-responses (**G**) for the non-mutated (*n* = 6) and mutated GlyR α3L^185L^ D141A/K (*n* = 5 each). (**H**, **I**) Representative patch clamp recordings from HEK293T cells expressing mutated GlyR α3L^185L^ E192A/K subjected to various glycine concentrations (in µM). (**J**, **K**) Quantification of the absolute maximum current amplitudes (**J**) and glycine dose-responses (**K**) for the non-mutated (*n* = 6) and mutated GlyR α3L^185L^ E192A/K (*n* = 6 and *n* = 7, respectively). (**L**, **M**) Representative patch clamp recordings from HEK293T cells expressing mutated GlyR α3L^185L^ D194A/K subjected to various glycine concentrations (in µM). (**N**, **O**) Quantification of the absolute maximum current amplitudes (**N**) and glycine dose-responses (**O**) for the non-mutated (*n* = 6) and mutated GlyR α3L^185L^ E192A/K (*n* = 5 each). Data are presented as mean ± SEM. Lines indicate dose-response-dependent non-linear regression. *p < 0.05.

The conformation of the recently postulated Cs^+^ binding site comprising D141, E192, and D194 was generated in VMD utilizing the structure of the human GlyR α3 bound with glycine and potentiator AM-3607^27^. Cs^+^ ions were inserted afterwards based on the recently published prediction^15^ and indicate its interplay with the addressed amino acid positions (Fig. 1A). As shown previously^15^, the current amplitude in non-mutated GlyR α3L^185L^ expressing cells increased after Cs^+^ application in a concentration-dependent manner (Fig. 1B; Table 1). We applied different concentrations of the agonist to cover the full range of GlyR responses (min, max) and to be able to derive the apparent affinity of the respective agonists (EC_50_).

**Table 1:** Absolute (in nA) and normalized (to 0.1 mM glycine) maximum current amplitude of HEK293T cells expressing non-mutated and mutated GlyR α3L^185L^ in response to 0.1 mM glycine and various Cs^+^ concentrations.

	0.1 mM glycine	0.5 mM Cs^+^	5 mM Cs^+^	50 mM Cs^+^	150 mM Cs^+^
GlyR α3L 185L (*n* = 12)					
Abs.	1.621 ± 0.300	0.143 ± 0.073	0.484 ± 0.111	2.007 ± 0.394	2.218 ± 0.332
Norm.	1	0.077 ± 0.019	0.351 ± 0.066	1.410 ± 0.237	1.766 ± 0.334
D141A (*n* = 16)					
Abs.	1.197 ± 0.175	0.066 ± 0.006	0.241 ± 0.036	1.399 ± 0.179	1.709 ± 0.219
Norm.	1	0.072 ± 0.011	0.226 ± 0.031	1.366 ± 0.202	1.663 ± 0.218
D141K (*n* = 11)					
Abs.	1.306 ± 0.256	0.035 ± 0.003	0.212 ± 0.035	1.596 ± 0.331	1.978 ± 0.298
Norm.	1	0.039 ± 0.009	0.213 ± 0.051	1.424 ± 0.263	1.940 ± 0.345
E192A (*n* = 11)					
Abs.	1.137 ± 0.255	0.093 ± 0.031	0.310 ± 0.078	1.356 ± 0.232	1.844 ± 0.237
Norm.	1	0.084 ± 0.015	0.290 ± 0.053	1.477 ± 0.260	2.297 ± 0.448
E192K (*n* = 22)					
Abs.	1.496 ± 0.136	0.146 ± 0.038	0.525 ± 0.098	1.448 ± 0.175	1.734 ± 0.213
Norm.	1	0.087 ± 0.014	0.322 ± 0.043	0.961 ± 0.071	1.175 ± 0.099
D194A (*n* = 11)					
Abs.	0.427 ± 0.099	0.042 ± 0.004	0.131 ± 0.022	0.559 ± 0.105	0.792 ± 0.151
Norm.	1	0.185 ± 0.059	0.510 ± 0.154	1.795 ± 0.455	2.763 ± 0.868
D194K (*n* = 12)					
Abs.	0.412 ± 0.108	0.073 ± 0.019	0.140 ± 0.021	0.263 ± 0.042	0.363 ± 0.050
Norm.	1	0.226 ± 0.032	0.489 ± 0.078	0.927 ± 0.148	1.347 ± 0.265

First, we assessed glycine because we wanted to compare the Cs^+^ responses with a sub-saturating glycine concentration. For this purpose, we applied increasing concentrations of glycine (0.01 µM up to 1000 µM) and measured the current responses. While both alanine and lysine mutations at D141 (Fig. 1C-G; Table 2) and E192 (Fig. 1H-K; Table 2) did not result in a change in maximal GlyR current amplitudes, compared to the non-mutated GlyR α3L^185L^ (Fig. 1C; Table 2), alanine and lysine mutations at D194 both lead to a decrease in maximal current amplitudes generated by the mutant GlyRs (Fig. 1L-O; Table 2, *p < 0.05).

**Table 2:** Absolute (in nA) and normalized (in percent) maximum current amplitudes of HEK293T cells expressing non-mutated and mutated GlyR α3L^185L^ in response to various glycine concentrations.

	0.01 µM glycine	1 µM glycine	10 µM glycine	100 µM glycine	500 µM glycine	1000 µM glycine
GlyR α3L 185L (*n* = 6)						
Abs.	0.026 ± 0.003	0.041 ± 0.011	0.141 ± 0.051	1.386 ± 0.198	1.593 ± 0.277	1.595 ± 0.225
Norm.	1.588 ± 0.186	2.429 ± 0.596	9.551 ± 3.918	80.685 ± 6.630	90.940 ± 3.200	92.970 ± 4.317
D141A (*n* = 5)						
Abs.	0.019 ± 0.003	0.033 ± 0.007	0.189 ± 0.114	1.521 ± 0.189	1.367 ± 0.247	1.399 ± 0.193
Norm.	1.264 ± 0.211	2.146 ± 0.589	10.960 ± 5.890	94.342 ± 2.384	82.992 ± 6.113	87.478 ± 7.381
D141K (*n* = 5)						
Abs.	0.019 ± 0.004	0.029 ± 0.008	0.043 ± 0.011	1.314 ± 0.164	1.280 ± 0.178	1.424 ± 0.130
Norm.	1.330 ± 0.316	1.916 ± 0.570	2.948 ± 0.877	85.083 ± 7.535	82.999 ± 9.826	92.776 ± 4.322
E192A (*n* = 6)						
Abs.	0.036 ± 0.010	0.030 ± 0.005	0.153 ± 0.036	1.120 ± 0.155	1.135 ± 0.237	1.192 ± 0.255
Norm.	3.382 ± 1.279	2.617 ± 0.512	12.711 ± 3.025	91.697 ± 5.048	88.870 ± 3.517	92.385 ± 4.761
E192K (*n* = 7)						
Abs.	0.036 ± 0.006	0.049 ± 0.011	0.277 ± 0.079	1.273 ± 0.220	1.621 ± 0.252	1.480 ± 0.207
Norm.	2.161 ± 0.331	2.895 ± 0.476	18.619 ± 8.587	76.3141 ± 2.055	90.434 ± 4.970	83.344 ± 4.024
D194A (*n* = 5)						
Abs.	0.023 ± 0.005	0.027 ± 0.005	0.047 ± 0.019	0.698 ± 0.177	0.619 ± 0.186	0.921 ± 0.298
Norm.	3.800 ± 1.225	5.368 ± 2.688	10.112 ± 5.403	82.121 ± 7.898	64.360 ± 4.419	88.036 ± 8.238
D194K (*n* = 5)						
Abs.	0.045 ± 0.005	0.059 ± 0.010	0.091 ± 0.024	0.538 ± 0.105	0.504 ± 0.091	0.563 ± 0.079
Norm.	7.692 ± 0.635	9.799 ± 0.679	15.244 ± 3.340	88.914 ± 8.079	82.983 ± 3.755	94.644 ± 3.280

**Fig. 2:**
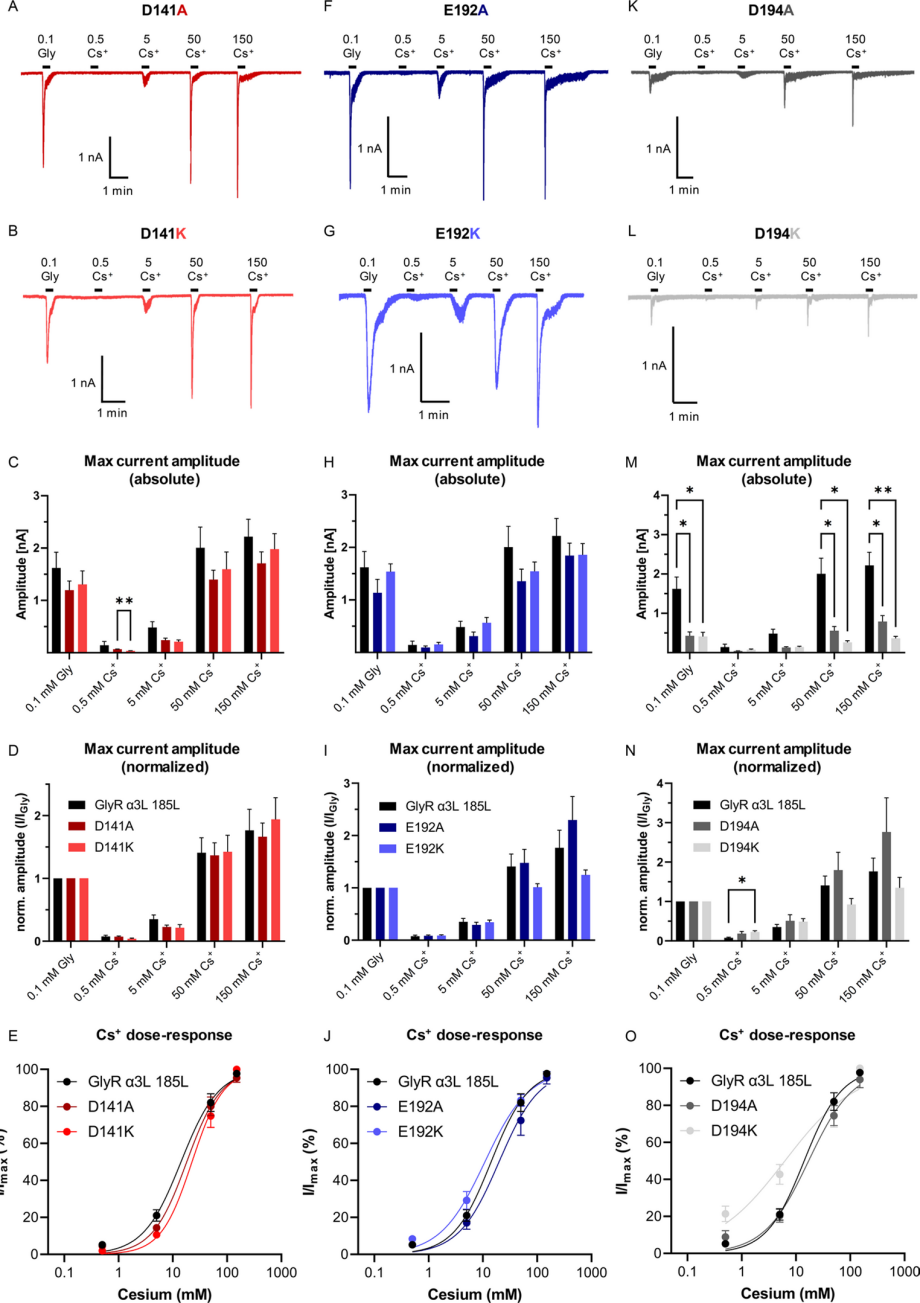
Effects of D141, E192, and D194 mutations on glycine- and Cs^+^-evoked GlyR α3L^185L^ currents. (**A**, **B**) Representative patch clamp recordings from HEK293T cells expressing the edited GlyR α3L^185L^ D141A/K mutants subjected to 0.1 mM glycine or various Cs^+^ concentrations (in mM). (**C**–**E**) Quantification of the absolute (**C**) and normalized maximum current amplitudes (**D**) as well as cesium dose-response curves (**E**) for the non-mutated (*n* = 12) and mutated GlyR α3L^185L^ D141A (*n* = 16), D141K (*n* = 11). (**F**, **H**) Representative patch clamp recordings from HEK293T cells expressing the edited GlyR α3L^185L^ E192A/K mutants subjected to 0.1 mM glycine or various Cs^+^ concentrations (in mM). (**H**–**J**) Quantification of the absolute (**H**) and normalized maximum current amplitudes (**I**) as well as cesium dose-response curves (**J**) for the non-mutated (*n* = 12) and mutated GlyR α3L^185L^ E192A (*n* = 11), E192K (*n* = 22). (**K**, **L**) Representative patch clamp recordings from HEK293T cells expressing the edited GlyR α3L^185L^ D194A/K mutants subjected to 0.1 mM glycine or various Cs^+^ concentrations (in mM). (**M**–**O**) Quantification of the absolute (**M**) and normalized maximum current amplitudes (N) as well as cesium dose-response curves (**O**) for the non-mutated (*n* = 12) and mutated GlyR α3L^185L^ D194A (*n* = 11), D194K (*n* = 12). Data are presented as mean ± SEM. Lines indicate dose-response-dependent non-linear regression. *p < 0.05, **p < 0.01.

Next, we investigated the role of D141, E192 and D194 in Cs^+^-evoked GlyR currents (Fig. 2). For the D141 mutations we found no significant differences to the GlyR α3L^185L^ regarding the activation by 0.1 mM glycine or the various Cs^+^ concentrations (Fig. 2A and E; Table 1). However, at 0.5 mM Cs^+^, the D141K channels produced significantly lower current amplitudes than the GlyR α3L^185L^ D141A (Fig. 2C; Table 1, **p < 0.01), but upon normalization to the glycine response no significant difference was evident (Fig. 2D; Table 2). Furthermore, dose-response curves for Cs^+^ indicated no apparent difference between the mutated and non-mutated GlyR channels (Fig. 2E; Table 3). Furthermore, GlyRs with E192 mutations exhibited responses to 0.1 mM glycine and Cs^+^ concentrations similarly to those of the non-mutated receptors (Fig. 2F and H; Table 1). Likewise, normalization of the maximum amplitude of Cs^+^-evoked currents to the currents evoked by glycine demonstrated no significant differences when compared to the non-mutated GlyR α3L^185L^ (Fig. 2I; Table 1). The dose-response curve for Cs^+^ also indicated no apparent difference in terms of EC_50_ and Hill coefficients between the mutated and the non-mutated GlyRs (Fig. 2J; Table 3). However, as already observed in response to 1 mM glycine (Table 4), we found a significant increase in the desensitization constant of E192K in response to 150 mM Cs^+^ when compared to the non-mutated GlyRs (Table 4), again indicating prolonged desensitization after activation. Thus, while mutations at D141 and E192 resulted only in slight changes of GlyR activity in response to glycine and Cs^+^, both alanine and lysine mutations at D194 lead to significant decrease in the maximum current amplitudes in response to 0.1 mM glycine as well as to 50 and 150 mM Cs^+^ compared to the non-mutated GlyR α3L^185L^ (Figs. 2 K-M, Table 1, *p < 0.05, **p < 0.01). However, normalization to the respective glycine-evoked currents abolished these effects almost completely, but the D194K channels exhibited a higher relative current response to 0.5 mM Cs^+^ (Fig. 2N; Table 1, *p < 0.05). While the EC_50_ and Hill slope of the dose-response curve for D194A showed no noteworthy difference to the non-mutated GlyR α3L^185L^, both the EC_50_ and Hill slope of D194K were found to be reduced (Fig. 2O; Table 3), indicating higher Cs^+^ potency and reduced cooperativity of Cs^+^ docking at multiple binding sites of the D194K GlyR variant. Taken together, amino acid position D194 obviously plays an important role in GlyR activation by both agonists, glycine and Cs^+^, with both D194A and D194K affecting maximal current amplitudes, and D194K selectively influencing Cs^+^-elicited GlyR currents.

**Table 3:** EC_50_ values and hill slope coefficients of dose-response curves of HEK293T cells expressing non-mutated and mutated GlyR α3L^185L^ in response to various glycine and Cs^+^ concentrations.

	Glycine		Cs^+^	
	EC_50_	Hill slope	EC_50_	Hill slope
GlyR α3L 185L	41.18 µM (*n* = 6)	1.446 (*n* = 6)	14.14 mM (*n* = 12)	1.253 (*n* = 12)
D141A	27.93 µM (*n* = 5)	1.989 (*n* = 5)	18.04 mM (*n* = 16)	1.370 (*n* = 16)
D141K	44.06 µM (*n* = 5)	1.948 (*n* = 5)	22.55 mM (*n* = 11)	1.515 (*n* = 11)
E192A	28.79 µM (*n* = 6)	1.770 (*n* = 6)	19.81 mM (*n* = 11)	1.167 (*n* = 11)
E192K	39.08 µM (*n* = 7)	0.930 (*n* = 7)	10.86 mM (*n* = 22)	1.063 (*n* = 22)
D194A	53.38 µM (*n* = 5)	0.748 (*n* = 5)	17.06 mM (*n* = 11)	1.058 (*n* = 11)
D194K	30.03 µM (*n* = 5)	1.380 (*n* = 5)	6.24 mM (*n* = 12)	0.655 (*n* = 12)

**Table 4:** Desensitization of non-mutated and mutated GlyR α3L^185L^ after 1 mM glycine and 150 mM Cs^+^ (Tau in s).

	1 mM glycine	150 mM Cs^+^
GlyR α3L 185L	2.287 ± 0.421 (*n* = 6)	0.963 ± 0.098 (*n* = 9)
D141A	2.115 ± 0.295 (*n* = 5)	1.342 ± 0.134 (*n* = 13)
D141K	1.815 ± 0.297 (*n* = 5)	1.025 ± 0.095 (*n* = 8)
E192A	1.582 ± 0.138 (*n* = 6)	0.946 ± 0.109 (*n* = 7)
E192K	3.716 ± 0.733 (*n* = 7)**	5.036 ± 0.778 (*n* = 17)****
D194A	1.846 ± 0.231 (*n* = 4)	0.819 ± 0.127 (*n* = 6)
D194K	2.878 ± 0.361 (*n* = 5)	1.566 ± 0.604 (*n* = 8)

Asterisks indicate significant statistic differences between E192A and E192K GlyR variants (glycine, **p < 0.01) or between the non-mutated GlyR α3L^185L^ and the E192K variant (Cs^+^, ****p < 0.0001).

**Fig. 3:**
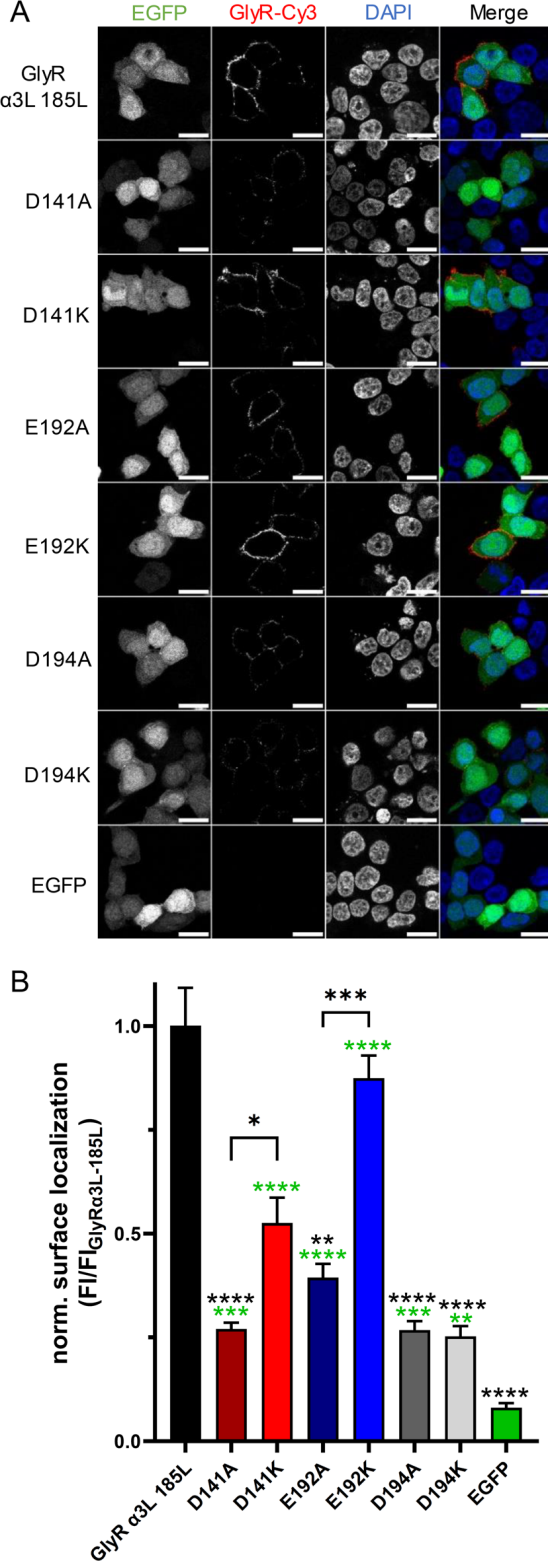
Analysis of cell surface localization of D141, E192 and D194 mutant GlyR α3L^185L^ proteins. (**A**) Representative images of HEK293T cells expressing EGFP or the HA-tagged GlyR α3L^185L^, as well as the D141, E192 and D194 GlyR mutants. EGFP fluorescence reveals transfected cells, DAPI the nucleus and the HA-tagged GlyRs were stained with antibodies carrying the Cy3 fluorophore. The scale bars represent 20 μm. (**B**) Quantification of the total Cy3 fluorescence in ratio to the cell number and normalized to the mean of the non-mutated GlyR α3L^185L^. This yielded the normalized GlyR surface location for EGFP (*n* = 36), HA-tagged GlyR α3L^185L^ (*n* = 46), D141A (*n* = 35), D141K (*n* = 37), E192A (*n* = 37), E192K (*n* = 41), D194A (*n* = 38) and D194K (*n* = 39) expression constructs. Data are presented as mean ± SEM. Green asterisks above the bars display significant differences compared to the EGFP control. Black asterisks indicate significant differences compared to the non-mutated GlyR α3L^185L^ or between GlyR mutants (brackets above the bars). *p < 0.05; ***p < 0.01; ***p < 0.001; ****p < 0.0001.

Many of the aforementioned differences of GlyR mutants, in particular regarding maximal current amplitudes, could be due to impairment of GlyR surface expression in HEK293T cells. Hence, to investigate whether the observed effects were due to changes in GlyR surface expression, we analyzed the surface localization of the non-mutated and mutated GlyRs using confocal microscopy (Fig. 3). Obviously, all constructs resulted in GlyR localization at the cell surface higher than that of the EGFP negative control (Fig. 3A; Table 5). However, the amount of receptor at the cell surface, normalized and compared to the non-mutated GlyR α3L^185L^, was significantly reduced for the D141A mutant (****p < 0.0001), the E192A mutant (**p < 0.0015), as well as the D194A (****p < 0.0001) and D194K mutants (****p < 0.0001, Fig. 3B; Table 5). Interestingly, the GlyR surface expression was significantly higher in the lysine mutants of D141 (*p = 0.05) and E192 (***p < 0.001) when compared to their corresponding alanine variants (Fig. 3B; Table 5). In fact, this effect was not observed when comparing the D194 lysine and alanine mutants (Fig. 3B; Table 5). Thus, all the alanine or lysine substitutions of D141, E192, or D194 reduce GlyR surface expression to different degrees, which does not correlate with the different responsiveness in terms of maximal current amplitudes of the mutant GlyRs to glycine or Cs^+^ and rules out the hypothesis that impaired surface expression of the GlyR mutants were responsible for the observed effects.

**Table 5:** Surface localization of non-mutated and mutated GlyR α3L^185L^ in HEK293T cells (in fluorescence per cell).

	Fluo./cell (a.u.)
GlyR α3L 185L (*n* = 46)	827.264 ± 75.460
D141A (*n* = 35)	223.554 ± 12.327
D141K (*n* = 37)	434.414 ± 50.607
E192A (*n* = 37)	325.876 ± 27.210
E192K (*n* = 41)	722.899 ± 45.281
D194A (*n* = 38)	221.439 ± 17.731
D194K (*n* = 39)	208.786 ± 20.700
GFP (*n* = 35)	67.860 ± 9.270

Altogether, mutations of the three investigated positions D141, E192, and D194 differentially affected agonist responses, with D141A/K or E192A/K mutants not significantly affecting receptor activation. However, the E192K mutant prolonged receptor desensitization following activation. In addition, the position D194 appears essential for activation of surface GlyR by glycine or Cs^+^, with a marginal but significant preference of D194K over non-mutated channels for Cs^+^-elicited currents at 0.5 mM.

## Discussion

In this study we investigated the molecular determinants of Cs^+^- and glycine-dependent GlyR activation. As published recently, molecular dynamic simulations revealed the moiety consisting of D141, E192 and D194 as a possible binding site for Cs^+^^15^. It is located near the editable site P185 and the loops that might contribute to the transduction of the glycine binding signal to the inner pore^5–7^. E192 and D194 are also associated with the binding of Zn^2+^ and Cu^2+^ to the GlyR, which can have opposing, modifying effects on glycine-evoked currents^17,18^. In order to test whether Cs^+^ binding is influenced by these sites, we mutated the GlyR α3L^185L^ at the aforementioned positions to code for the neutral amino acid alanine or the positively charged lysine. After performing whole-cell patch clamp electrophysiology, our analyses of the maximum amplitudes in response to various glycine and Cs^+^ concentrations showed that the mutations differentially affected glycine- and Cs^+^-evoked responses. D141A/K or E192A/K had no effect, while D194A/K reduced both glycine- and Cs^+^-evoked current responses, with a marginal but significant preference of D194K over non-mutated channels for Cs^+^-evoked currents at 0.5 mM. Likewise, the apparent Cs^+^ affinity (EC_50_) and Hill slope (cooperativity) were reduced, indicating higher affinity and negative cooperativity, respectively. Finally, desensitization was prolonged in E192K expressing cells in response to application of saturating glycine and cesium concentrations.

The results showed that mutations of D141 had no considerable effect on both glycine and Cs^+^-evoked currents. Even though it is located in the highly conserved, receptor family name-given Cys-loop, both mutations did not change the concentration-dependent response to Cs^+^ or glycine. Even though the maximal current amplitude was reduced at the lowest Cs^+^ concentration (0.5 mM) in cells expressing D141K, compared to D141A, normalization to glycine-evoked current responses put this apparent difference into perspective. Thus, D141 does not seem to be directly involved in signal transduction of Cs^+^. This conclusion is in line with previous research demonstrating that, within the Cys-loop, only residues such as L142, F145, P146 and D148 play a role in gating^28^.

E192 and D194 are located on the β-sheet 9, that precedes the C-loop (β9-β10), part of the orthosteric glycine binding site and follows the F-loop (β8-β9), which contributes to the transduction of the gating signal to the transmembrane domains (TM) forming the inner pore^6,7,16^. Alterations of the β9 sheet may disturb the positioning of these loops, possibly influencing receptor efficacy and kinetics through changes in agonist binding or signal transduction. In addition, these residues might interact with the flexible C-terminus that follows the TM4^19^.

Although the E192 mutants did not affect the evoked currents, E192K exhibited a prolonged desensitization following activation by high glycine and cesium concentrations. This position was proposed to be relevant for modulation of GlyR currents, either potentiating them after Zn^2+^ or inhibiting them after Cu^2+^ binding^17–19^. Prior mutational analyses demonstrated that E192A mutation ablated potentiation by Zn^2+^ via alteration of the backbone structure^20^. This feature appears to be less critical for glycine- or cesium-dependent GlyR activation. Surprisingly, the replacement of the side chain charge in the E192K channel seems to influence desensitization after application of glycine and particularly cesium. Since desensitization is primarily associated with rearrangements in the TMs^29^, the potential interactions with the post-TM4 region could impact it. However, no effect on desensitization was previously observed in the GlyR α1 E192 mutants^20^, which could be due to its shorter C-terminus compared to GlyR α3. In another perspective, the positive charge at this position might mimic Zn^2+^ binding, which was proposed to stabilize the closed C-loop conformation^19^, maybe delaying the signal of persistent activation.

While amino acid substitutions at D141 and E192 positions had no considerable effect on glycine- or Cs^+^-evoked maximal currents, both D194A and D194K substitutions resulted in a significant reduction. This might be correlated to an altered C- or F-loop arrangement, resulting in reduced general receptor activation. Prior research of GlyR α1 D194A indicated no effect on the maximum current but displayed an increased EC_50_ for glycine^20^. The difference to our results might be attributed to the edited site, which elevates glycine and cesium apparent affinity^8,9,15^, or it may be due to subunit-specific differences. Specifically, potential interactions with the TM4 might play a role as it was previously recognized to contribute to disparities in agonist efficiency between GlyR α1 and α3^30^. This interaction might explain the higher sensitivity of GlyR α3 to the potentiating effects of Zn^2+^ when it binds at this position^19^. The observed decrease in EC_50_ for cesium of the D194K mutant should be considered carefully, as saturation was not reached. However, the mutation could potentially set up a long-range effect on the other potential cesium binding sites above the orthosteric glycine binding moiety. This might be similar to how the involvement of D194 in Zn^2+^ binding affects the C-loop conformation^19^. In conjunction with the considerable disparity in the Hill coefficients for glycine and cesium, these findings underscore a potential interaction between D194 with cesium. In fact, the lysine substitution at position D194 might mimic the positive charge of Cs^+^ and thereby facilitate GlyR conductance evoked with 0.5 mM Cs^+^, triggering negative cooperativity through inhibiting binding at different sites. Moreover, despite their direct adjacency, the intriguing differences in GlyR desensitization and activation between the E192K and D194K mutants require additional investigations to elucidate the underlying mechanisms.

D194A and D194K mutants equally reduced the amplitudes of glycine- and Cs^+^-evoked currents, but this observation could have been due to reduced cell surface expression of the GlyR D194 mutants. It was previously shown that neutral or positively charged amino acids in the F loop reduce the surface expression of mutated GlyRs^16^. While all the mutants were impaired in GlyR surface expression, as revealed by our confocal microscopy analysis of HA surface-stained GlyR mutants, alanine substitutions at positions D141 and E192 had a more pronounced effect on GlyR surface expression than the corresponding lysine substitutions. As D141A and D194A/K variants were similarly impaired in GlyR surface expression, but D141A channels were not impaired in glycine- or Cs^+^-dependent GlyR activation, our patch clamp results emphasize the impact of D194A/K substitutions with regard to activation of cell surface-expressed GlyR α3^185L^.

Taken together, our study confirms previous reports on the relevance of the F-loop in GlyR activation and regulation of sensitivity to agonists or allosteric modulators in general^7–9,16,18,21^ but it also demonstrates in particular that a negative charge at position 194 is required for efficacious agonist-dependent activation of GlyR α3L^185L^ with the nearby position 192 affecting receptor desensitization. Furthermore, it supports the hypothesis that evolutionary conservation and diversification of the glycine binding domain from *Escherichia coli* to vertebrates involves the F-loop and its association with plasma membrane phospholipids in eukaryotes^31^ as critical sites for agonistic and allosteric regulation of GlyR activation.

## Supplementary Information

Below is the link to the electronic supplementary material.

Supplementary Material 1

## Data Availability

All data generated or analysed during this study are included in its supplementary information files.
